# Alterations in Neurotrophins in Alcohol-Addicted Patients during Alcohol Withdrawal

**DOI:** 10.3390/brainsci14060583

**Published:** 2024-06-06

**Authors:** Magda Malewska-Kasprzak, Maria Skibińska, Monika Dmitrzak-Węglarz

**Affiliations:** 1 Department of Psychiatry, Poznan University of Medical Sciences, 61-701 Poznan, Poland; m.malew@poczta.onet.pl; 2 Department of Psychiatric Genetics, Poznan University of Medical Sciences, 61-701 Poznan, Poland; mweglarz@ump.edu.pl

**Keywords:** alcohol use disorder, alcohol withdrawal syndrome, delirium tremens, neurotrophic factors

## Abstract

Background: Alcohol use disorder (AUD) is related to mental and somatic disorders that result in alcohol withdrawal syndrome (AWS), with 30% of AWS cases leading to life-threatening delirium tremens (DTs). Currently, studies do not support using any one biomarker in DTs. Neurotrophins affect neuromodulation, playing a role in the pathogenesis of AUD, AWS, and DTs. Methods: This review aims to summarize experimental and clinical data related to neurotrophins and S100B in neuroplasticity, as well as neurodegeneration in the context of AUD, AWS, and DTs. This work used publications that were selected based on the protocol consistent with the Preferred Reporting Items for Systematic Reviews and Meta-Analysis (PRISMA) statement. Results: The BDNF level could be a good candidate biomarker for relapse susceptibility, as it is significantly reduced during consumption and gradually increases during abstinence. GDNF influences AUD through its integral role in the function of dopaminergic neurons and ablates the return to alcohol-drinking behavior. NGF protects neurons from ethanol-induced cytotoxic damage and affects recovery from cognitive deficits after brain damage. The NT-3 level is decreased after alcohol exposure and is involved in compensatory mechanisms for cognitive decline in AUD. NT-4 affects oxidative stress, which is associated with chronic alcohol consumption. S100B is used as a biomarker of brain damage, with elevated levels in serum in AUD, and can protect 5-HT neurons from the damage caused by alcohol. Conclusions: BDNF, GDNF, NT-3, NT-4, NGF, and S100B may be valuable markers for withdrawal syndrome. In particular, the most relevant is their association with the development of delirium complications. However, there are few data concerning some neurotrophins in AWS and DTs, suggesting the need for further research.

## 1. Introduction

### Alcohol Use Disorder (AUD), Alcohol Withdrawal Syndrome (AWS), and Delirium Tremens (DTs)

Alcohol use disorder (AUD) is associated with behavioral, cognitive, and physiological symptoms, including a strong need to consume alcohol, decreased control over use, use despite harmful consequences, social impairment, risky use patterns, and physiologic tolerance and withdrawal [1,2,3]. AUD is the most commonly diagnosed addiction. Overall, 12% of the Polish population abuse alcohol, with 2% considered AUD, and over 16% of the population drink alcohol at risk, more in males than in females (4.1% vs. 0.4%) [4]. Only 20% of AUD patients are treated in their lifetime [1]. Research shows that people with AUD are at risk of somatic disorders, including organic brain damage; liver disease and alcoholic polyneuropathy; and other psychiatric disorders such as anxiety, depression, and bulimia [3,5].

Alcohol withdrawal syndrome (AWS) is a life-threatening complication of AUD occurring in approximately 30% of patients. Symptoms of AWS include handshaking, headaches, nausea, vomiting, dry mouth, accelerated heart rate, hypertension, dilated pupils, feeling anxiety, irritability, excessive sweating, insomnia, depressive states, seizures, and delirium [6,7], which appear on the third or fourth day after stopping alcohol consumption. In each case of AWS, it is advisable to perform laboratory tests to assess electrolyte disturbances, dehydration, leukocytosis, liver dysfunction, and electroencephalogram (EEG). The mortality of AWS is up to 37% without appropriate treatment [8]. Unfortunately, so far, no useful biomarkers have been explored to facilitate the diagnosis and treatment of AWS.

AUD and subsequent AWS and DTs are involved in disturbances of neuroplasticity and neurodegeneration [9], leading to cognitive impairment with white matter (WM) atrophy, axonal loss, and demyelination at the frontal lobe and hippocampus [10,11]. Neurotrophic growth factors are involved in neuronal plasticity during AWS. Neurotrophins are soluble proteins released by neurons into the intercellular space and have a chemotropic effect. They include (1) nerve growth factor (NGF), (2) brain-derived neurotrophic factor (BDNF), (3) neurotrophine-3 (NT-3), (4) neurotrophine-4/5 (NT-4/5), and (5) glial cell line-derived neurotrophic factor (GDNF). In addition to neurotrophins, the survival of neurons is influenced by neurotrophic proteins (neurotrophic factors), e.g., the fibroblast growth factor (FGF) [12,13]. BDNF is a part of the brain’s nerve growth factor (NGF)-related family of neurotrophic factors., while GDNF is a member of the transforming growth factor (TGF-β) line of factors [14]. NGF, BDNF, and GDNF activate tyrosine kinase A (TrkA), tyrosine kinase B (TrkB), and tyrosine kinase RET. The primary role of neurotrophins is to participate in neurogenesis at several levels [15]. They participate in regulating neuronal survival, differentiation, and growth processes [16]. They also participate in the development of neuronal plasticity and facilitate synaptic transport. These mechanisms ensure proper memory and regeneration processes. The synthesis of appropriate neurotrophins occurs in transforming precursor proteins (pro-neurotrophins) and may occur with the participation of nerve cells and other cells. In turn, S100B protein belongs to the family of small-molecule calcium-binding proteins and is a recognized biomarker used in the diagnosis of central nervous system damage of various etiologies [17]. The involvement of neurotrophic factors in alcoholism is currently an important area of multidisciplinary experimental and clinical research. Here, we aimed to review actual experimental and clinical data related to neurotrophins, S100B participation in neuroplasticity, and neurodegeneration in the context of AUD, AWS, and DTs.

## 2. Materials and Methods

This work used publications that were selected based on the protocol consistent with the Preferred Reporting Items for Systematic Reviews and Meta-Analysis (PRISMA) statement. This study was not registered on Prospero. In the first step, we selected online repositories of citations from biomedical literature, such as PubMed, EMBASE, and Web of Science Core Research. The selected databases provide optimal search as a minimum requirement, according to Bramer et al. (2017) [18]. Articles in these databases were searched automatically based on the following given keywords: [(neurotrophin) AND [(alcohol use disorder) OR (alcohol withdrawal syndrome) OR (delirium tremens)]. Duplicate records were automatically removed using the ZOTERO bibliography manager. Further selection of records was carried out based on the inclusion and exclusion criteria.

The inclusion criteria included (a) full-text articles, (b) published in English, (c) published in peer-reviewed journals, (d) human studies, (e) original articles, and (f) meta-analyses.

The exclusion criteria included (a) non-human studies, (b) genetic studies (SNP genotyping or GWAS), (c) comorbidity, (d) other diagnoses, (e) research on children and adolescents, (f) retracted articles, (g) non-English articles, and (h) duplicates.

**Figure 1:**
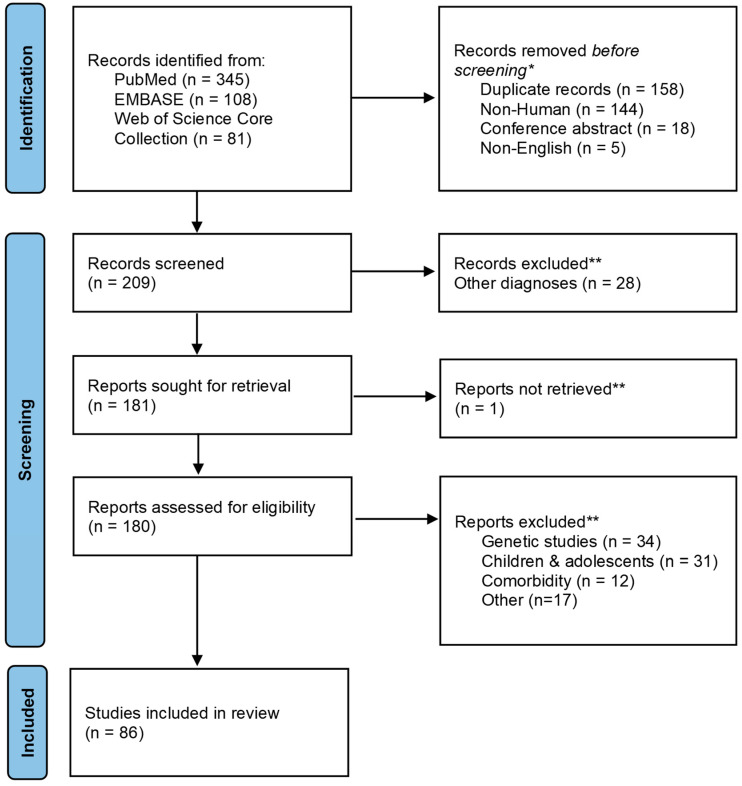
Publication selection process.* Record excluded using automated tools built-in database and bibliography manager. ** Record excluded by a researcher.

Because the PubMed database found the most significant number of relevant articles, it was manually searched for individual neurotrophins such as BDNF, GDNF, NGF, NT-3, NT-4, and S100B. Selected publications that met the above-described criteria were subjected to a full-text review of the remaining records. Figure 1 presents schematically the publication selection process. (Figure 1).

## 3. Results

### 3.1. Neurotrophins in AUD

#### 3.1.1. Brain-Derived Neurotrophic Factor (BDNF)

BDNF regulates important physiological and pathological functions of the body, influencing the development and growth of neurons, learning and memory processes, apoptosis, neurogenesis, and neuroregeneration [19,20,21,22,23,24]. In addition to the CNS, BDNF is also located in the heart, skeletal muscles, smooth muscle cells, lungs, blood platelets, and fibroblasts. The biological effect of BDNF occurs by activating TrkB and p75NTR receptors [25,26,27]. Animal studies revealed that alcohol consumption decreases BDNF levels in the hippocampus and increases TrkB and p75 receptors in the frontal cortex [28,29]. Moreover, BDNF could be a good candidate biomarker for relapse susceptibility AUD. Results have shown a significant reduction in BDNF serum levels in AUD patients [30,31,32,33,34,35]. However, in another study, there was no significant difference in plasma BDNF levels in AUD patients and social drinkers groups [36]. Moreover, serum BDNF levels were negatively correlated with average drinks per drinking day [37]. Other results suggest neurotrophin signaling deficits of BDNF are associated with alcohol-induced cognitive impairment [38,39]. In AUD, peripheral BDNF levels are also related to the presence of depressive symptoms [40,41]. Findings support the role of the BDNF in the pathophysiology of schizophrenia [40,42,43], PTSD [44,45,46,47], bipolar disorder [48,49,50], suicide attempts [51,52], vulnerability to stress [53,54,55], and anxiety disorder [56]. Moreover, hazardous alcohol users revealed either significantly lower or significantly higher BDNF levels compared to then non-hazardous people living with HIV [57].

Alterations in BDNF levels might play a role in the inheritance of AUD [58,59], related to AUD pathophysiology [60,61,62]. DNA methylation signatures and the signal nucleotide polymorphism of candidate genes are the underlying factors in the role of BDNF in addiction [63,64,65]. Val66Met BDNF gene polymorphism was associated with a higher risk [66], the earlier occurrence of relapse among patients treated for AUD [67], higher alcohol consumption [68], and the severity of smoking in AUD [69,70]. The A allele containing 66Met promotes BDNF expression, and this may protect humans against CVD induced by long-term excessive alcohol intake [71]. Moreover, offspring from families with multiple cases of alcohol dependence have a greater likelihood of developing AUD that is caused by an interaction between allelic variation in GABRA2 and BDNF genes [72]. BDNF Val66Met polymorphism was also involved in lower reward dependence scores in the offspring of alcohol-dependent adult female probands [73]. It may contribute to alcohol dependence vulnerability via lower impairment of executive function performance [74]. However, in other studies, genotype and allele distributions of the BDNF gene polymorphism did not differ significantly between alcoholic and control subjects [75,76,77].

#### 3.1.2. Glial Cell Line-Derived Neurotrophic Factor (GDNF)

GDNF is produced by glial cells, mainly astrocytes and Schwann cells. GDNF is expressed in the striatum, thalamus, cortex, and hippocampus [78]. GDNF activates the receptor tyrosine kinase RET, requiring the presence of the coreceptor GDNF-family receptor α1 (GFRα1) [79]. Moreover, GDNF triggers dopaminergic neurons in the midbrain, in both nigrostriatal and mesolimbic projections. The infusion of GDNF into the ventral tegmental area increases dopamine release in the noradrenaline [80]. GDNF expression in the striatum improves dopaminergic transmission [81] and is related to psychiatric conditions, such as depression, anxiety, stress, schizophrenia [82], and addictive behaviors [83]. GDNF also influences AUD through its role in the regulation of dopaminergic neurons. Chronic, intermittent alcohol use leads to decreased endogenous, whereas acute alcohol exposure triggers the upregulation of GDNF expression in the mesolimbic system. Ford et al. (2023) infused adeno-associated virus encoding human glial-derived neurotrophic factor (AAV2-hGDNF). GDNF expression stopped the return to alcohol consumption during a period of repeated abstinence–alcohol reintroduction challenges, which is associated with dopamine signaling in the nucleus accumbens (NA) [84]. Moreover, GDNF serum levels were lower in AUD and were negatively associated with alcohol tolerance [85].

#### 3.1.3. Nerve Growth Factor (NGF)

In the 1950s, Levi-Montalcini and Cohen discovered the first neurotrophy of NGF, for which they received the Nobel Prize in 1986. NGF consists of two alpha, one beta, and two gamma subunits, as well as one or two zinc ions. It occurs in both the central and peripheral nervous system. It also affects cells outside the nervous system: the eyeball and the skin. Moreover, NGF has a protective effect on the cholinergic system [86,87]. It is involved in the development and differentiation of nerve cells and preventing damage to mature neurons. NGF is also known for its beneficial effect on recovery from cognitive deficits after brain damage [88]. In addition, it could play an essential role in protecting neurons from cytotoxic damage induced by ethanol [13]. NGF is a candidate marker for the early stages of alcohol misuse [89]. The plasma NGF concentrations in alcohol-dependent patients were significantly lower than in the controls [90]. Increased levels of NGF with a negative correlation in alcohol-dependent patients may play a role in the regeneration of damage incurred by chronic alcohol use [91]. In humans, the Neuregulin 1 (NRG1) genotype is related to substance use, and this relationship is modulated by adverse life events, with a gain-of-function allele being protective [92]. Moreover, the Neuregulin 3 (NRG3)/Erb-B2 receptor tyrosine kinase 4 (ERBB4) signaling pathway participates in nicotine disorder. NRG3, activated after nicotine intake, binds to ERBB4 and causes GABA release [93].

#### 3.1.4. Neurotrophin-3 (NT-3)

Neurotrophin-3 (NT-3) was discovered in 1990 by Jones et al. NT-3 promotes neurons’ growth, maturation, survival, and function [94]. In ethanol-exposed rats, BDNF and NT-3 plasma levels decreased, and there was a negative correlation between hippocampal BDNF mRNA levels and recognition memory [95]. Moreover, NT-3 and BDNF may compensate for cognitive decline in the early stages of AUD but not in later phases [35].

#### 3.1.5. Neurotrophin-3 (NT-4)

Neurotrophin-4 (NT-4) is a part of the neurotrophin family that depends on the tropomyosin receptor kinase B (TrkB). NT-4 confers neuroprotective effects following cerebral ischemia [96]. The role of NT-4 in the development of AUD has not yet been clarified. There is an association between NT-4 signaling and the deleterious effects of alcohol, namely through oxidative stress. Ethanol metabolism is related to the formation of the reduced form of NADH+. It is delivered to mitochondria, where during the Q-cycle, reactive oxygen species (ROS) are formed due to electron leakage. The level of NADH increases due to ethanol metabolism, leading to an increase in ROS production, which is related to neurotrophin signaling [97].

#### 3.1.6. S100 Calcium-Binding Protein B (S100B)

**Figure 2:**
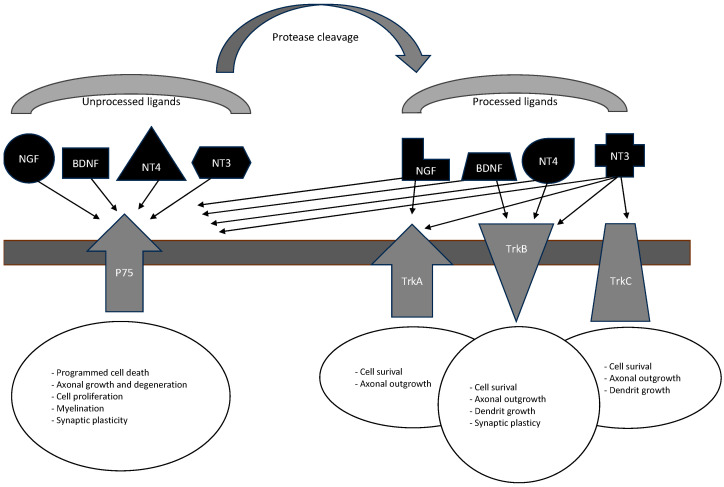
Biological effects of neurotrophins. NGF—nerve growth factor; BDNF—brain-derived neurotrophic factor; NT-3 and 4—neurotrophin-3 and -4; P75—neurotrophin receptor p75; TrkA, B, and C—tyrosine kinase receptor type A, B, and C.

The term S100B refers to a protein identified in the mid-1960s from brain extracts that is characterized by solubility in a 100% saturated solution with ammonium sulfate [98]. S100B is used as a biomarker of brain damage [99,100], mechanical brain injury, traumatic brain injury induced by alcoholic intoxication, and long-term chemical external impacts (prolonged consumption of ethanol or other psychoactive addictive substances), as well as complex biochemical degenerative processes (associated with Parkinson and Alzheimer’s diseases) [101]. Khandare et al. revealed a correlation of S100B levels (along with neuron-specific enolase protein) with the severity of the infract in ischemic stroke, pointing to S100B as a predictive marker protein for the assessment of how strong the ischemic strike is and what clinical prognosis may be expected. S100B is a protein that can protect 5-HT neurons from the damage caused by alcohol [102,103]. In AUD patients, S100B protein levels have been widely found to be elevated in blood serum and astrocytes [104]. Figure 2 presents the biological effect of neurotrophins.

### 3.2. Neurotrophins in AWS

#### 3.2.1. Brain-Derived Neurotrophic Factor (BDNF)

BDNF levels in AUD patients are initially significantly reduced during consumption and gradually increase during abstinence [28,105]. However, a study on Polish patients did not verify this general picture. In AUD patients, after the clinical symptoms of alcohol withdrawal syndrome (AWS) had subsided, BDNF levels did not differ significantly between the BDNF values before and after the presence of AWS [106]. Other findings indicated an evolution of BDNF concentrations after AWS [107], which was associated with withdrawal severity [85]. Although research confirms that AWS is associated with changes in BDNF levels, the diversity of study results emphasizes the need for further research. Moreover, changes in BDNF levels and liver stiffness after alcohol withdrawal are related to changes in homeostatic mechanisms [107]. The results also show an association between BDNF expression and the symptomatology of AWS, which is related to changes in the methylation of the BDNF IV gene promoter [108]. In AUD and crack-cocaine use disorder (CUD), changes in mRNA expression in peripheral blood lymphocytes were observed after detoxification treatment by performing real-time PCR and examining its association with frontal assessment battery performance [109].

#### 3.2.2. Glial Cell Line-Derived Neurotrophic Factor (GDNF)

A study revealed lower GDNF serum levels in AUD patients during abstinence [110]. Heberlin et al. indicated that serum levels of GDNF were significantly decreased in AUD patients and were negatively associated with alcohol tolerance. GDNF serum levels were negatively associated with AWS [85]. Moreover, Maier et al. showed the epigenetic regulation of GDNF after ethanol consumption and AWS [111]. These results hold clinical relevance since differences in GDNF mRNA expression and DNA methylation could be a target for pharmacological interventions [111]. Koskela et al. studied the role of GDNF on alcohol-seeking behavior in mice. mRNA levels in NA were more than four times the levels in mice after drinking alcohol compared to the control group. This suggests that an increase in GDNF expression upon alcohol drinking is related to the activation of another mesolimbic reward pathway [112]. 

#### 3.2.3. Nerve Growth Factor (NGF)

Studies have indicated a correlation between plasma NGF levels and AWS. Lee et al. showed that plasma NGF levels were elevated in AUD patients within 24 h of abstinence [91]. Köhler et al. indicated lower NGF levels in AUD patients after acute withdrawal over two weeks of alcohol abstinence compared to the control group. The NGF level initially increased and then significantly decreased from days 3 to 14 [105]. Moreover, decreased NGF concentrations in patients suffering from AUD, which stabilize after AWS, are in line with neurological risk factors and AWS symptoms. It has been suggested that plasma NGF levels regulate mechanism that counteracts alcohol intoxication [105]. These findings are related to the epigenetic downregulation of the NGF gene during alcohol withdrawal [105,113,114]. Moreover, there is a correlation between alterations in serum NGF concentrations and changes in the methylation of the NGF promoter during AWS. AUD patients exhibited a decrease in serum NGF levels from day 7 to day 14 after the presence of AWS and a significant increase in the methylation of the CpG sites within the NGF gene promoter [114]. Moreover, there is a linear association between the methylation of CpG sites within the NGF gene promoter and IL-6 serum levels [65]. The connections between inflammatory and neurotrophic factors may have implications for neuroadaptive changes during recovery from AUD [115].

#### 3.2.4. S100 Calcium-Binding Protein B (S100B)

Liappas et al. measured S100B serum levels in patients with AWS during hospitalization and indicated a significantly increased level of S100B at admission compared to discharge after approximately 4–5 weeks. S100B levels were different in AUD patients with either mild or high alcohol consumption over a period of up to one year before assessment. A good correlation was found between the release of S100B and the global functioning scale [104]. Girad et al. described changes in serum S100B levels that were increased significantly during abstinence in alcohol-dependent subjects after one month of withdrawal and then plateaued, regardless of abstinence status at six months [116]. It is important to note how the S100B protein manifests in various stages of addiction to alcohol: in the course of the disease and during withdrawal and treatment phases. These data suggest a possible use of S100B measurements in detecting AUD patients with high alcohol consumption and monitoring the alcohol detoxification treatment. Moreover, S100B was found to decline rapidly in AWS. S100B could be relevant in the neurobiology of AWS. It may be indirectly related to glutamatergic activity and the stress level during AWS [117].

### 3.3. Neurotrophins in Delirium

There are few data concerning neurotrophins in the context of delirium tremens. Malewska et al. indicated higher BDNF levels after the presence of AWS with DTs than before AWS [28]. The findings suggest that BDNF might be a candidate for personalized medicine-based detection of delirium [118]. Moreover, Huang et al. suggested that chronic drinking leads to decreased BDNF levels, and patients with more deficient BDNF expression are prone to the development of DTs [119]. Additionally, BDNF levels are elevated after prompt alcohol detoxification treatment. These findings suggest that BDNF can modify the neuroadaptive processes of AUD and the phenotypes of AWS [119]. In the case of postoperative delirium, there was no difference in BDNF levels relative to delirium status. However, the percent decline in BDNF was more significant in patients who developed delirium than in those who did not, which suggests that plasma BDNF levels may be a biomarker for delirium [120]. In oncology, a cross-sectional relationship has been established between blood BDNF and TNF-α levels with delirium. The association between cancer and reduced serum BDNF levels may be mediated by confounding factors [121].

S100B levels were positively associated with postoperative delirium [122]. Patients with postoperative delirium showed an increase in S100B levels on the first postoperative day [98]. In obstetric patients, the rise in S100B levels was approximately three times greater in those who developed delirium than in those who did not. It is a more specific predictor of delirium [123]. However, S100B levels on admission did not predict delirium in affected elderly patients [124]. Moreover, mDNA in the neurotrophic genes of GDNF in both the brain and blood increases with age, especially among delirium patients [125]. Serum NGF levels were significantly elevated in AUD patients, especially in those with prior delirium. The presented situation reflects the activity of NGF as an endogenous repair mechanism for damaged neurons [126]. Changes in neurotrophin levels in AUD, AWS, and DTs are shown in Table 1.

## 4. Future Direction

DTs are involved in disturbances of neuroplasticity and neurodegeneration [9], leading to white matter (WM) atrophy, axonal loss, and demyelination [10,11]. Studies do not currently support using any one biomarker in DTs. Neurotrophic growth factors are involved in neuronal plasticity during DTs. Future studies could benefit from including other diagnostic modalities, such as EEG [127]. Recent advances in clinical chemistry have revealed novel approaches for the specific detection of AUD through assays of phosphatidyl ethanol (PEth) or ethyl glucuronide (EtG), carbohydrate-deficient transferrin (CDT), and unique ethanol metabolites [128]. Other in vitro findings indicate the effect of ethanol and acetaldehyde on the level of peripheral oxidative stress markers—products of oxidative modification of lipids (lipid peroxidation products), DNA (8-hydroxy-2-deoxyguanosine, 8-OHdG), and proteins (protein carbonyls) in blood plasma. There are changes in these parameters and the activity of antioxidant enzymes (SOD and catalase) in patients with AUD. Studies indicate that, at a particular stage of the disease, oxidative stress could play a protective rather than pathogenic role in the body [129]. There are also several genes related to DTs [130]. Epigenetic analysis is an attractive option for detecting delirium in clinical practice. Research points to the influence of DNA methylation on the expression of genes encoding for pro-inflammatory cytokines in delirium patients [131]. The connection of DNA methylation with cholinergic synapse is also relevant [125]. Specific markers were associated with promising results and require future studies. Some biomarkers are significant in some clinical settings, such as comorbidities. These may provide direction for future studies. Heterogeneity across study methods could be connected with inconclusive results. The standardization of clinical assessment methods will provide more clarity in the results. These factors require further investigation. However, the main form of treatment for alcoholism is psychotherapy, which allows for the determination of the specific characteristics of alcoholism (e.g., Lesch Type III). The differences in the mechanisms of AUD between sexes are also relevant. Female AUD patients suffer more severely from dysfunctional interpersonal relationships than their male AUD counterparts [132]. Table 1 summarizes the potential roles of these neurotrophins in the context of AWS and DTs, highlighting their association with the development of delirium and other complications. Future research should focus on these neurotrophins to enhance our understanding and improve clinical outcomes for patients suffering from AUD.

Various factors can affect the results of studies, including laboratory errors (e.g., the selection, processing, and storage of materials; reagents; and equipment used), statistical power, quality control, confounding factors, selection bias, and fundamental differences due to interactions and effect modifiers that can provide information on trait and disease mechanisms. It is difficult to determine what precisely is the cause in the works cited. The reliably obtained results in an individual study should be regarded as a preliminary report. When more studies are conducted, a specific common direction of change can be traced in review papers, and verification can be carried out using meta-analyses. The latter allows us to draw more robust conclusions.

## 5. Conclusions

### 5.1. Brain-Derived Neurotrophic Factor (BDNF)

In AUD patients, peripheral BDNF levels have been observed to significantly decrease. This reduction is linked to the neurotoxic effects of chronic alcohol consumption, which impair neurogenesis and neuronal plasticity. Interestingly, BDNF levels tend to increase during periods of abstinence, suggesting its potential role as a biomarker for monitoring relapse susceptibility. These dynamic changes in BDNF levels reflect the brain’s attempt to recover and adapt during abstinence, making BDNF a promising candidate for tracking treatment progress and predicting relapse risk in AUD patients.

In AWS, BDNF levels continue to demonstrate significant variability. While some studies indicate a further reduction during the acute withdrawal phase, others suggest an initial dip followed by a gradual increase as the body begins to recover. This variability underscores the complexity of the withdrawal process and the brain’s adaptive responses.

In cases of DTs, the most severe form of AWS, BDNF levels have not been extensively studied. However, the severe neuropsychiatric symptoms associated with DTs imply a potential dysregulation of BDNF, which warrants further investigation. Understanding BDNF dynamics in DTs can provide insights into the pathophysiology of this life-threatening condition and aid in developing targeted therapeutic strategies.

### 5.2. Glial Cell Line-Derived Neurotrophic Factor (GDNF)

Peripheral GDNF levels are also impacted by chronic alcohol consumption and withdrawal. In AUD patients, GDNF levels are generally lower, which correlates with the neurodegenerative effects of long-term alcohol exposure and its impact on dopaminergic pathways. This reduction in GDNF may contribute to the persistence of addictive behaviors and difficulty in achieving sustained abstinence.

Studies have shown that GDNF levels can increase during withdrawal, potentially as a compensatory mechanism to counteract the neurotoxic effects of alcohol and support neuronal survival and recovery. This adaptive response highlights GDNF’s role in the brain’s repair processes during withdrawal.

In DTs, the data on GDNF levels remain sparse. Given GDNF’s involvement in dopaminergic regulation and neuroprotection, it is plausible that severe fluctuations or deficiencies in GDNF could exacerbate the neuropsychiatric symptoms observed in DTs. Further research is necessary to elucidate the precise role of GDNF in DTs and its potential as a biomarker for identifying individuals at risk of developing this severe condition.

### 5.3. Nerve Growth Factor (NGF), Neurotrophin-4 (NT-3), and Neurotrophin-4 (NT-4)

NGF offers neuroprotection against ethanol-induced cytotoxic damage and aids in the recovery from cognitive deficits post-brain injury. Similarly, NT-3 levels decrease following alcohol exposure and are involved in compensatory mechanisms for cognitive decline associated with AUD. NT-4 impacts oxidative stress, which is linked to chronic alcohol consumption.

### 5.4. S100 Calcium-Binding Protein B (*S100B*)

S100B, a marker of glial activation and brain damage, showed elevated levels in the serum of individuals with AUD. This elevation is indicative of the chronic neuroinflammatory state and glial activation resulting from prolonged alcohol exposure. High S100B levels reflect ongoing neurodegeneration and may correlate with cognitive deficits and other neuropsychiatric symptoms seen in AUD.

During alcohol withdrawal, S100B levels typically rise, reflecting the acute neuroinflammatory response and glial activation as the brain reacts to the sudden absence of alcohol. These elevated levels can serve as a biomarker for the severity of withdrawal symptoms and the extent of brain damage incurred during prolonged alcohol use.

In DTs, S100B levels are expected to be significantly elevated due to the intense neuroinflammatory response and widespread neuronal damage characteristic of this condition. Monitoring S100B levels in patients experiencing DTs could provide critical information regarding the severity of brain injury and guide therapeutic interventions to mitigate neurotoxicity and improve clinical outcomes.

### 5.5. Summary

The peripheral levels of neurotrophins offer valuable insights into the neurobiological underpinnings of AUD, AWS, and DTs. BDNF serves as a potential biomarker for relapse susceptibility and recovery during abstinence. GDNF’s role in dopaminergic regulation and neuroprotection highlights its importance in adaptive responses to alcohol withdrawal. S100B, as a marker of glial activation and brain damage, is an indicator of the neuroinflammatory response and neuronal injury in these conditions. Collectively, these neurotrophins could be crucial in developing biomarkers for the diagnosis, monitoring, and treatment of AUD and its associated withdrawal symptoms. However, the current body of research presents limited data on the role of some neurotrophins in AWS and DTs, indicating a need for further studies. Continued exploration in this area is essential to uncover reliable biomarkers and develop targeted interventions for managing and mitigating the effects of AUD and its severe withdrawal symptoms. 

## Figures and Tables

**Table 1:** Changes in neurotrophin levels in AUD, AWS, and DTs.

Neurotrophins	AUD	AWS	DTs
NGF	↓ [85] ↑ [91,126]	?	↑ [126]
BDNF	↓ [30,32,33,34,35], ↔ [36]	↑ [28,105]	↑ [28] ↓ [118]
GDNF	↓ [85]	↓ [85,110]	↑ [125]
NT-3, NT-4	?	?	?

AUD—alcohol use disorder; AWS—alcohol withdrawal syndrome; DTs—delirium tremens; NGF—nerve growth factor; BDNF—brain-derived neurotrophic factor; GDNF—glial cell line-derived neurotrophic factor; NT-3—neurotrophin-3; NT-4—neurotrophin-4/5. ?—direction of changes not determined, ↑—increased concentration, ↓—decreased concentration, ↔—no change in concentration

## Data Availability

Not applicable.

## References

[1] Wolf C., Curry A., Nacht J., Simpson S.A. (2020). Management of Alcohol Withdrawal in the Emergency Department: Current Perspectives. Open Access Emerg. Med. OAEM.

[2] Lieberman R., Jensen K.P., Clinton K., Levine E.S., Kranzler H.R., Covault J. (2020). Molecular Correlates of Topiramate and GRIK1 Rs2832407 Genotype in Pluripotent Stem Cell–Derived Neural Cultures. Alcohol. Clin. Exp. Res..

[3] Crews F.T., Boettiger C.A. (2009). Impulsivity, Frontal Lobes and Risk for Addiction. Pharmacol. Biochem. Behav..

[4] Zgliczyński W. (2016). Alkohol w Polsce (Alcohol in Poland). INFOS.

[5] Salize H.J., Dillmann-Lange C., Stern G., Kentner-Figura B., Stamm K., Rössler W., Henn F. (2002). Alcoholism and Somatic Comorbidity among Homeless People in Mannheim, Germany. Addict. Abingdon Engl..

[6] Smith A.H., Ovesen P.L., Skeldal S., Yeo S., Jensen K.P., Olsen D., Diazgranados N., Zhao H., Farrer L.A., Goldman D. (2018). Risk Locus Identification Ties Alcohol Withdrawal Symptoms to SORCS2. Alcohol. Clin. Exp. Res..

[7] Cavallazzi R., Saad M., Marik P.E. (2012). Delirium in the ICU: An Overview. Ann. Intensive Care.

[8] Rahman A., Paul M. (2024). Delirium Tremens. StatPearls.

[9] Jung M.E., Metzger D.B. (2010). Alcohol Withdrawal and Brain Injuries: Beyond Classical Mechanisms. Mol. Basel Switz..

[10] Harper C., Matsumoto I. (2005). Ethanol and Brain Damage. Curr. Opin. Pharmacol..

[11] Hill S.Y., Wang S., Kostelnik B., Carter H., Holmes B., McDermott M., Zezza N., Stiffler S., Keshavan M.S. (2009). Disruption of Orbitofrontal Cortex Laterality in Offspring from Multiplex Alcohol Dependence Families. Biol. Psychiatry.

[12] Neurotrofiny—Aktualny Stan Wiedzy Postępy Psychiatrii i Neurologii. http://www.ppn.ipin.edu.pl/archiwum/neurotrofiny-aktualny-stan-wiedzy.html.

[13] Lee T.-I., Yang C.-S., Fang K.-M., Tzeng S.-F. (2009). Role of Ciliary Neurotrophic Factor in Microglial Phagocytosis. Neurochem. Res..

[14] Airaksinen M.S., Saarma M. (2002). The GDNF Family: Signalling, Biological Functions and Therapeutic Value. Nat. Rev. Neurosci..

[15] Wang T.-Y., Lee S.-Y., Chang Y.-H., Chen S.-L., Chen P.S., Chu C.-H., Huang S.-Y., Tzeng N.-S., Lee I.H., Chen K.C. (2018). Correlation of Cytokines, BDNF Levels, and Memory Function in Patients with Opioid Use Disorder Undergoing Methadone Maintenance Treatment. Drug Alcohol Depend..

[16] Lee R., Kermani P., Teng K.K., Hempstead B.L. (2001). Regulation of Cell Survival by Secreted Proneurotrophins. Science.

[17] Rajewska-Rager A., Pawlaczyk M. (2016). The Role of S100B Protein as a Potential Marker in Affective Disorders. Psychiatr. Pol..

[18] Bramer W.M., Rethlefsen M.L., Kleijnen J., Franco O.H. (2017). Optimal Database Combinations for Literature Searches in Systematic Reviews: A Prospective Exploratory Study. Syst. Rev..

[19] Zhang X.Y., Tan Y.-L., Chen D.-C., Tan S.-P., Yang F.-D., Zunta-Soares G.B., Soares J.C. (2016). Effects of Cigarette Smoking and Alcohol Use on Neurocognition and BDNF Levels in a Chinese Population. Psychopharmacology.

[20] Tsai S.-J., Liao D.-L., Yu Y.W.-Y., Chen T.-J., Wu H.-C., Lin C.-H., Cheng C.-Y., Hong C.-J. (2005). A Study of the Association of (Val66Met) Polymorphism in the Brain-Derived Neurotrophic Factor Gene with Alcohol Dependence and Extreme Violence in Chinese Males. Neurosci. Lett..

[21] Zanardini R., Fontana A., Pagano R., Mazzaro E., Bergamasco F., Romagnosi G., Gennarelli M., Bocchio-Chiavetto L. (2011). Alterations of Brain-Derived Neurotrophic Factor Serum Levels in Patients with Alcohol Dependence. Alcohol. Clin. Exp. Res..

[22] Mansur R.B., Santos C.M., Rizzo L.B., Asevedo E., Cunha G.R., Noto M.N., Pedrini M., Zeni-Graiff M., Cordeiro Q., Vinberg M. (2016). Brain-Derived Neurotrophic Factor, Impaired Glucose Metabolism, and Bipolar Disorder Course. Bipolar Disord..

[23] Bus B., Molendijk M., Penninx B., Buitelaar J., Kenis G., Prickaerts J., Elzinga B., Voshaar R. (2011). Determinants of Serum Brain-Derived Neurotrophic Factor. Psychoneuroendocrinology.

[24] Barbey A.K., Colom R., Paul E., Forbes C., Krueger F., Goldman D., Grafman J. (2014). Preservation of General Intelligence Following Traumatic Brain Injury: Contributions of the Met66 Brain-Derived Neurotrophic Factor. PLoS ONE.

[25] Zhou L., Xiong J., Ruan C.-S., Ruan Y., Liu D., Bao J.-J., Zhou X.-F. (2018). ProBDNF/p75NTR/Sortilin Pathway Is Activated in Peripheral Blood of Patients with Alcohol Dependence. Transl. Psychiatry.

[26] Fernandez G., Lew B., Stewart W., Savage L. (2015). Behavioral and Neural Consequences of Chronic versus Binge Ethanol Treatment: A Profile of Rodent Models of Adoeslcent and Adult Ethanol Exposure. Alcohol. Clin. Exp. Res..

[27] Shin S., Stewart R., Ferri C.P., Kim J.-M., Shin I.-S., Kim S.-W., Yang S.-J., Yoon J.-S. (2010). An Investigation of Associations between Alcohol Use Disorder and Polymorphisms on ALDH2, BDNF, 5-HTTLPR, and MTHFR Genes in Older Korean Men. Int. J. Geriatr. Psychiatry.

[28] Malewska-Kasprzak M., Permoda-Pachuta A., Skibińska M., Malinowska-Kubiak M., Rybakowski F., Dmitrzak-Węglarz M. (2024). Investigation of Serum BDNF Levels in Alcohol Withdrawal Syndrome with and without Other Medical Co-Morbidities. Alcohol.

[29] Chen J., Hutchison K.E., Calhoun V.D., Claus E.D., Turner J.A., Sui J., Liu J. (2015). CREB-BDNF Pathway Influences Alcohol Cue-Elicited Activation in Drinkers. Hum. Brain Mapp..

[30] Zanardini R., Fontana A., Gennarelli M., Bocchio- Chiavetto L. (2010). Alterations of Brain-Derived Neurotrophic Factor Serum Levels in Alcohol Dependence Patients. Eur. Neuropsychopharmacol..

[31] Mo M., Fu X.-Y., Zhang X.-L., Zhang S.-C., Zhang H.-Q., Wu L., Li J.-L., Zhou L. (2021). Association of Plasma Pro-Brain-Derived Neurotrophic Factor (proBDNF)/Mature Brain-Derived Neurotrophic Factor (mBDNF) Levels with BDNF Gene Val66Met Polymorphism in Alcohol Dependence. Med. Sci. Monit. Int. Med. J. Exp. Clin. Res..

[32] García-Marchena N., Silva-Peña D., Martín-Velasco A.I., Villanúa M.Á., Araos P., Pedraz M., Maza-Quiroga R., Romero-Sanchiz P., Rubio G., Castilla-Ortega E. (2017). Decreased Plasma Concentrations of BDNF and IGF-1 in Abstinent Patients with Alcohol Use Disorders. PLoS ONE.

[33] Kauer-Sant’Anna M., Tramontina J., Andreazza A.C., Cereser K., da Costa S., Santin A., Yatham L.N., Kapczinski F. (2007). Traumatic Life Events in Bipolar Disorder: Impact on BDNF Levels and Psychopathology. Bipolar Disord..

[34] Xu Y.-Y., Ge J.-F., Chen J., Liang J., Pang L.-J., Gao W.-F., Cao Y., Shan F., Liu Y., Yan C.-Y. (2020). Evidence of a Relationship Between Plasma Leptin, Not Nesfatin-1, and Craving in Male Alcohol-Dependent Patients After Abstinence. Front. Endocrinol..

[35] Requena-Ocaña N., Araos P., Flores M., García-Marchena N., Silva-Peña D., Aranda J., Rivera P., Ruiz J.J., Serrano A., Pavón F.J. (2021). Evaluation of Neurotrophic Factors and Education Level as Predictors of Cognitive Decline in Alcohol Use Disorder. Sci. Rep..

[36] D’Sa C., Dileone R.J., Anderson G.M., Sinha R. (2012). Serum and Plasma Brain-Derived Neurotrophic Factor (BDNF) in Abstinent Alcoholics and Social Drinkers. Alcohol.

[37] Portelli J., Farokhnia M., Deschaine S.L., Battista J.T., Lee M.R., Li X., Ron D., Leggio L. (2020). Investigating the Link between Serum Concentrations of Brain-Derived Neurotrophic Factor and Behavioral Measures in Anxious Alcohol-Dependent Individuals. Alcohol.

[38] García-Marchena N., Pizarro N., Pavón F.J., Martínez-Huélamo M., Flores-López M., Requena-Ocaña N., Araos P., Silva-Peña D., Suárez J., Santín L.J. (2020). Potential Association of Plasma Lysophosphatidic Acid (LPA) Species with Cognitive Impairment in Abstinent Alcohol Use Disorders Outpatients. Sci. Rep..

[39] Requena N., Silva-Peña D., García-Marchena N., Flores M., Rubio G., Rivera P., Pavon J., Suárez J., Torre R.D.L., Serrano A. (2023). Role for BDNF and Oleoylethanolamide (OEA) in Cognitive Impairment Associated with Alcohol Use Disorders in Humans and Preclinical Models. Alcohol.

[40] Umene-Nakano W., Yoshimura R., Ikenouchi-Sugita A., Hori H., Hayashi K., Ueda N., Nakamura J. (2009). Serum Levels of Brain-Derived Neurotrophic Factor in Comorbidity of Depression and Alcohol Dependence. Hum. Psychopharmacol..

[41] Buttenschøn H.N., Demontis D., Kaas M., Elfving B., Mølgaard S., Gustafsen C., Kaerlev L., Petersen C.M., Børglum A.D., Mors O. (2015). Increased Serum Levels of Sortilin Are Associated with Depression and Correlated with BDNF and VEGF. Transl. Psychiatry.

[42] Zai C.C., Manchia M., Zai G.C., Woo J., Tiwari A.K., de Luca V., Kennedy J.L. (2018). Association Study of BDNF and DRD3 Genes with Alcohol Use Disorder in Schizophrenia. Neurosci. Lett..

[43] Cheah S.-Y., Lawford B.R., Young R.M., Connor J.P., Phillip Morris C., Voisey J. (2014). BDNF SNPs Are Implicated in Comorbid Alcohol Dependence in Schizophrenia but Not in Alcohol-Dependent Patients without Schizophrenia. Alcohol.

[44] Neupane S.P., Bramness J.G., Lien L. (2017). Comorbid Post-Traumatic Stress Disorder in Alcohol Use Disorder: Relationships to Demography, Drinking and Neuroimmune Profile. BMC Psychiatry.

[45] Hemmings S.M.J., Martin L.I., Klopper M., van der Merwe L., Aitken L., de Wit E., Black G.F., Hoal E.G., Walzl G., Seedat S. (2013). BDNF Val66Met and DRD2 Taq1A Polymorphisms Interact to Influence PTSD Symptom Severity: A Preliminary Investigation in a South African Population. Prog. Neuropsychopharmacol. Biol. Psychiatry.

[46] Jeon H.J., Kang E.-S., Lee E.H., Jeong E.-G., Jeon J.-R., Mischoulon D., Lee D. (2012). Childhood Trauma and Platelet Brain-Derived Neurotrophic Factor (BDNF) after a Three Month Follow-up in Patients with Major Depressive Disorder. J. Psychiatr. Res..

[47] Hernaus D., van Winkel R., Gronenschild E., Habets P., Kenis G., Marcelis M., van Os J., Myin-Germeys I., Collip D. (2014). Brain-Derived Neurotrophic Factor/FK506-Binding Protein 5 Genotype by Childhood Trauma Interactions Do Not Impact on Hippocampal Volume and Cognitive Performance. PLoS ONE.

[48] Jacoby A.S., Munkholm K., Vinberg M., Pedersen B.K., Kessing L.V. (2016). Cytokines, Brain-Derived Neurotrophic Factor and C-Reactive Protein in Bipolar I Disorder—Results from a Prospective Study. J. Affect. Disord..

[49] Jiang X., Xu K., Hoberman J., Tian F., Marko A.J., Waheed J.F., Harris C.R., Marini A.M., Enoch M.-A., Lipsky R.H. (2005). BDNF Variation and Mood Disorders: A Novel Functional Promoter Polymorphism and Val66Met Are Associated with Anxiety but Have Opposing Effects. Neuropsychopharmacology.

[50] Mansur R.B., Santos C.M., Rizzo L.B., Cunha G.R., Asevedo E., Noto M.N., Pedrini M., Zeni M., Cordeiro Q., McIntyre R.S. (2016). Inter-Relation between Brain-Derived Neurotrophic Factor and Antioxidant Enzymes in Bipolar Disorder. Bipolar Disord..

[51] Ropret S., Zupanc T., Komel R., Paska A.V. (2015). Investigating the Associations between Polymorphisms in the NTRK2 and NGFR Genes and Completed Suicide in the Slovenian Sample. Psychiatr. Genet..

[52] Berent D., Szymańska B., Kulczycka-Wojdala D., Macander M., Pawłowska Z., Wojnar M. (2020). The Role of Childhood Adversities, FKBP5, BDNF, NRN1, and Generalized Self-Efficacy in Suicide Attempts in Alcohol-Dependent Patients. Pharmacol. Rep. PR.

[53] Kim T.Y., Kim S.J., Chung H.G., Choi J.H., Kim S.H., Kang J.I. (2017). Epigenetic Alterations of the BDNF Gene in Combat-Related Post-Traumatic Stress Disorder. Acta Psychiatr. Scand..

[54] Sharma S., Graham R., Rohde R., Ceballos N.A. (2017). Stress-Induced Change in Serum BDNF Is Related to Quantitative Family History of Alcohol Use Disorder and Age at First Alcohol Use. Pharmacol. Biochem. Behav..

[55] Guillot C.R., Kelly M.E., Phillips N.B., Su M.-Y., Douglas M.E., Poe D.J., Berman M.E., Liang T. (2023). BDNF and Stress/Mood-Related Interactions on Emotional Disorder Symptoms, Executive Functioning, and Deliberate Self-Harm. J. Psychiatr. Res..

[56] Gorka S.M., Teppen T., Radoman M., Phan K.L., Pandey S.C. (2020). Human Plasma BDNF Is Associated With Amygdala-Prefrontal Cortex Functional Connectivity and Problem Drinking Behaviors. Int. J. Neuropsychopharmacol..

[57] Míguez-Burbano M.J., Espinoza L., Whitehead N.E., Bryant V.E., Vargas M., Cook R.L., Quiros C., Lewis J.E., Deshratan A. (2014). Brain Derived Neurotrophic Factor and Cognitive Status: The Delicate Balance among People Living with HIV, with and without Alcohol Abuse. Curr. HIV Res..

[58] Joe K.-H., Kim Y.-K., Kim T.-S., Roh S.-W., Choi S.-W., Kim Y.-B., Lee H.-J., Kim D.-J. (2007). Decreased Plasma Brain-Derived Neurotrophic Factor Levels in Patients with Alcohol Dependence. Alcohol. Clin. Exp. Res..

[59] Valerio A.G., Ornell F., Roglio V.S., Scherer J.N., Schuch J.B., Bristot G., Pechansky F., Kapczinski F., Kessler F.H.P., von Diemen L. (2022). Increase in Serum Brain-Derived Neurotrophic Factor Levels during Early Withdrawal in Severe Alcohol Users. Trends Psychiatry Psychother..

[60] Forero D.A., López-León S., Shin H.D., Park B.L., Kim D.-J. (2015). Meta-Analysis of Six Genes (BDNF, DRD1, DRD3, DRD4, GRIN2B and MAOA) Involved in Neuroplasticity and the Risk for Alcohol Dependence. Drug Alcohol Depend..

[61] Su N., Zhang L., Fei F., Hu H., Wang K., Hui H., Jiang X.F., Li X., Zhen H.N., Li J. (2011). The Brain-Derived Neurotrophic Factor Is Associated with Alcohol Dependence-Related Depression and Antidepressant Response. Brain Res..

[62] Bohnsack J.P., Teppen T., Kyzar E.J., Dzitoyeva S., Pandey S.C. (2019). The lncRNA BDNF-AS Is an Epigenetic Regulator in the Human Amygdala in Early Onset Alcohol Use Disorders. Transl. Psychiatry.

[63] Shen W., Liu H., Xie X., Liu H., Zhou W. (2017). Biochemical Diagnosis in Substance and Non-Substance Addiction. Adv. Exp. Med. Biol..

[64] Zhang H., Ozbay F., Lappalainen J., Kranzler H.R., van Dyck C.H., Charney D.S., Price L.H., Southwick S., Yang B.-Z., Rasmussen A. (2006). Brain Derived Neurotrophic Factor (BDNF) Gene Variants and Alzheimer’s Disease, Affective Disorders, Posttraumatic Stress Disorder, Schizophrenia, and Substance Dependence. Am. J. Med. Genet. Part B Neuropsychiatr. Genet. Off. Publ. Int. Soc. Psychiatr. Genet..

[65] Heberlein A., Schuster R., Kleimann A., Groh A., Kordon A., Opfermann B., Lichtinghagen R., Gröschl M., Kornhuber J., Bleich S. (2017). Joint Effects of the Epigenetic Alteration of Neurotrophins and Cytokine Signaling: A Possible Exploratory Model of Affective Symptoms in Alcohol-Dependent Patients?. Alcohol Alcohol..

[66] Enoch M.-A., White K.V., Waheed J., Goldman D. (2008). Neurophysiological and Genetic Distinctions between Pure and Comorbid Anxiety Disorders. Depress. Anxiety.

[67] Wojnar M., Brower K.J., Strobbe S., Ilgen M., Matsumoto H., Nowosad I., Sliwerska E., Burmeister M. (2009). Association between Val66Met Brain-Derived Neurotrophic Factor (BDNF) Gene Polymorphism and Post-Treatment Relapse in Alcohol Dependence. Alcohol. Clin. Exp. Res..

[68] Klimkiewicz A., Mach A., Jakubczyk A., Klimkiewicz J., Wnorowska A., Kopera M., Fudalej S., Burmeister M., Brower K., Wojnar M. (2017). COMT and BDNF Gene Variants Help to Predict Alcohol Consumption in Alcohol-Dependent Patients. J. Addict. Med..

[69] Pivac N., Nedic Erjavec G., Sagud M., Nikolac Perkovic M., Tudor L., Uzun S., Kovacic Petrovic Z., Konjevod M., Dvojkovic A., Kozumplik O. (2022). The Association between BDNF C270T Genetic Variants and Smoking in Patients with Mental Disorders and in Healthy Controls. Prog. Neuropsychopharmacol. Biol. Psychiatry.

[70] Jamal M., Van der Does W., Penninx B.W.J.H. (2015). Effect of Variation in BDNF Val(66)Met Polymorphism, Smoking, and Nicotine Dependence on Symptom Severity of Depressive and Anxiety Disorders. Drug Alcohol Depend..

[71] Serý O., Sťastný F., Zvolský P., Hlinomazová Z., Balcar V.J. (2011). Association between Val66Met Polymorphism of Brain-Derived Neurotrophic Factor (BDNF) Gene and a Deficiency of Colour Vision in Alcohol-Dependent Male Patients. Neurosci. Lett..

[72] Hill S.Y., Wang S., Carter H., Tessner K., Holmes B., McDermott M., Zezza N., Stiffler S. (2011). Cerebellum Volume in High-Risk Offspring from Multiplex Alcohol Dependence Families: Association with Allelic Variation in GABRA2 and BDNF. Psychiatry Res..

[73] Benzerouk F., Gierski F., Raucher-Chéné D., Ramoz N., Gorwood P., Kaladjian A., Limosin F. (2015). Association Study between Reward Dependence and a Functional BDNF Polymorphism in Adult Women Offspring of Alcohol-Dependent Probands. Psychiatr. Genet..

[74] Benzerouk F., Gierski F., Gorwood P., Ramoz N., Stefaniak N., Hübsch B., Kaladjian A., Limosin F. (2013). Brain-Derived Neurotrophic Factor (BDNF) Val66Met Polymorphism and Its Implication in Executive Functions in Adult Offspring of Alcohol-Dependent Probands. Alcohol.

[75] Matsushita S., Kimura M., Miyakawa T., Yoshino A., Murayama M., Masaki T., Higuchi S. (2004). Association Study of Brain-Derived Neurotrophic Factor Gene Polymorphism and Alcoholism. Alcohol. Clin. Exp. Res..

[76] Muschler M.A.N., Heberlein A., Frieling H., Vogel N., Becker C.-M., Kornhuber J., Bleich S., Hillemacher T. (2011). Brain-Derived Neurotrophic Factor, Val66Met Single Nucleotide Polymorphism Is Not Associated with Alcohol Dependence. Psychiatr. Genet..

[77] Grzywacz A., Samochowiec A., Ciechanowicz A., Samochowiec J. (2010). Family-Based Study of Brain-Derived Neurotrophic Factor (BDNF) Gene Polymorphism in Alcohol Dependence. Pharmacol. Rep. PR.

[78] Ortega-de San Luis C., Pascual A. (2016). Simultaneous Detection of Both GDNF and GFRα1 Expression Patterns in the Mouse Central Nervous System. Front. Neuroanat..

[79] Airavaara M., Planken A., Gäddnäs H., Piepponen T.P., Saarma M., Ahtee L. (2004). Increased Extracellular Dopamine Concentrations and FosB/DeltaFosB Expression in Striatal Brain Areas of Heterozygous GDNF Knockout Mice. Eur. J. Neurosci..

[80] Wang R., Li Y.-H., Xu Y., Li Y.-B., Wu H.-L., Guo H., Zhang J.-Z., Zhang J.-J., Pan X.-Y., Li X.-J. (2010). Curcumin Produces Neuroprotective Effects via Activating Brain-Derived Neurotrophic Factor/TrkB-Dependent MAPK and PI-3K Cascades in Rodent Cortical Neurons. Prog. Neuropsychopharmacol. Biol. Psychiatry.

[81] Grondin R., Littrell O.M., Zhang Z., Ai Y., Huettl P., Pomerleau F., Quintero J.E., Andersen A.H., Stenslik M.J., Bradley L.H. (2019). GDNF Revisited: A Novel Mammalian Cell-Derived Variant Form of GDNF Increases Dopamine Turnover and Improves Brain Biodistribution. Neuropharmacology.

[82] Ibáñez C.F., Andressoo J.-O. (2017). Biology of GDNF and Its Receptors—Relevance for Disorders of the Central Nervous System. Neurobiol. Dis..

[83] Liran M., Rahamim N., Ron D., Barak S. (2020). Growth Factors and Alcohol Use Disorder. Cold Spring Harb. Perspect. Med..

[84] Ford M.M., George B.E., Van Laar V.S., Holleran K.M., Naidoo J., Hadaczek P., Vanderhooft L.E., Peck E.G., Dawes M.H., Ohno K. (2023). GDNF Gene Therapy for Alcohol Use Disorder in Male Non-Human Primates. Nat. Med..

[85] Heberlein A., Muschler M., Wilhelm J., Frieling H., Lenz B., Gröschl M., Kornhuber J., Bleich S., Hillemacher T. (2010). BDNF and GDNF Serum Levels in Alcohol-Dependent Patients during Withdrawal. Prog. Neuropsychopharmacol. Biol. Psychiatry.

[86] Aloe L. (2011). Rita Levi-Montalcini and the Discovery of NGF, the First Nerve Cell Growth Factor. Arch. Ital. Biol..

[87] Reynolds P.M., Mueller S.W., MacLaren R. (2015). A Comparison of Dexmedetomidine and Placebo on the Plasma Concentrations of NGF, BDNF, GDNF, and Epinephrine during Severe Alcohol Withdrawal. Alcohol.

[88] Ledda R., Battagliese G., Attilia F., Rotondo C., Pisciotta F., Gencarelli S., Greco A., Fiore M., Ceccanti M., Attilia M.L. (2019). Drop-out, Relapse and Abstinence in a Cohort of Alcoholic People under Detoxification. Physiol. Behav..

[89] Lhullier A.C., Moreira F.P., da Silva R.A., Marques M.B., Bittencourt G., Pinheiro R.T., Souza L.D.M., Portela L.V., Lara D.R., Jansen K. (2015). Increased Serum Neurotrophin Levels Related to Alcohol Use Disorder in a Young Population Sample. Alcohol. Clin. Exp. Res..

[90] Yoon S.-J., Roh S., Lee H., Lee J.-Y., Lee B.-H., Kim Y.-K., Kim D.-J. (2006). Possible Role of Nerve Growth Factor in the Pathogenesis of Alcohol Dependence. Alcohol. Clin. Exp. Res..

[91] Lee B.C., Choi I.-G., Kim Y.-K., Ham B.-J., Yang B.-H., Roh S., Choi J., Lee J.-S., Oh D.-Y., Chai Y.-G. (2009). Relation between Plasma Brain-Derived Neurotrophic Factor and Nerve Growth Factor in the Male Patients with Alcohol Dependence. Alcohol.

[92] Vaht M., Laas K., Kiive E., Parik J., Veidebaum T., Harro J. (2017). A Functional Neuregulin-1 Gene Variant and Stressful Life Events: Effect on Drug Use in a Longitudinal Population-Representative Cohort Study. J. Psychopharmacol. Oxf. Engl..

[93] Kara H.G., Erdal M.E., Yılmaz S.G., Şengül C., Şengül C.B., Karakülah K. (2021). Association of NRG3 and ERBB4 Gene Polymorphism with Nicotine Dependence in Turkish Population. Mol. Biol. Rep..

[94] Arabska J., Łucka A., Strzelecki D., Wysokiński A. (2018). In Schizophrenia Serum Level of Neurotrophin-3 (NT-3) Is Increased Only If Depressive Symptoms Are Present. Neurosci. Lett..

[95] Silva-Peña D., García-Marchena N., Alén F., Araos P., Rivera P., Vargas A., García-Fernández M.I., Martín-Velasco A.I., Villanúa M.Á., Castilla-Ortega E. (2019). Alcohol-Induced Cognitive Deficits Are Associated with Decreased Circulating Levels of the Neurotrophin BDNF in Humans and Rats. Addict. Biol..

[96] Wang T., Zhang J., Li P., Ding Y., Tang J., Chen G., Zhang J.H. (2020). NT-4 Attenuates Neuroinflammation via TrkB/PI3K/FoxO1 Pathway after Germinal Matrix Hemorrhage in Neonatal Rats. J. Neuroinflamm..

[97] Heaton M.B., Moore D.B., Paiva M., Madorsky I., Mayer J., Shaw G. (2003). The Role of Neurotrophic Factors, Apoptosis-Related Proteins, and Endogenous Antioxidants in the Differential Temporal Vulnerability of Neonatal Cerebellum to Ethanol. Alcohol. Clin. Exp. Res..

[98] Michetti F., Clementi M.E., Di Liddo R., Valeriani F., Ria F., Rende M., Di Sante G., Romano Spica V. (2023). The S100B Protein: A Multifaceted Pathogenic Factor More Than a Biomarker. Int. J. Mol. Sci..

[99] Thaler H.W., Schmidsfeld J., Pusch M., Pienaar S., Wunderer J., Pittermann P., Valenta R., Gleiss A., Fialka C., Mousavi M. (2015). Evaluation of S100B in the Diagnosis of Suspected Intracranial Hemorrhage after Minor Head Injury in Patients Who Are Receiving Platelet Aggregation Inhibitors and in Patients 65 Years of Age and Older. J. Neurosurg..

[100] Gelabert-Gonzälez M., Aran-Echabe E., Serramito-García R. (2013). S-100B Protein and Chronic Subdural Hematoma. Front. Neurol..

[101] Martín-González C., Romero-Acevedo L., Fernández-Rodríguez C.M., Medina-Vega L., García-Rodríguez A., Ortega-Toledo P., González-Navarrete L., Vera-Delgado V.E., González-Reimers E. (2021). Brain-Derived Neurotrophic Factor among Patients with Alcoholism. CNS Spectr..

[102] Eriksen J.L., Druse M.J. (2001). Astrocyte-Mediated Trophic Support of Developing Serotonin Neurons: Effects of Ethanol, Buspirone, and S100B. Brain Res. Dev. Brain Res..

[103] Wu S.-Y., Chen C.-Y., Huang T.-L., Tsai M.-C. (2020). Brain-Derived Neurotrophic Factor and Glutathione Peroxidase as State Biomarkers in Alcohol Use Disorder Patients Undergoing Detoxification. Medicine.

[104] Liappas I., Tzavellas E.O., Kariyannis C., Piperi C., Schulpis C., Papassotiriou I., Soldatos C.R. (2006). Effect of Alcohol Detoxification on Serum S-100B Levels of Alcohol-Dependent Individuals. Vivo Athens Greece.

[105] Köhler S., Klimke S., Hellweg R., Lang U.E. (2013). Serum Brain-Derived Neurotrophic Factor and Nerve Growth Factor Concentrations Change after Alcohol Withdrawal: Preliminary Data of a Case-Control Comparison. Eur. Addict. Res..

[106] Ciszowski K., Gomółka E., Gawlikowski T., Szpak D., Potoczek A., Boba M. (2016). Brain-Derived Neurotrophic Factor (BDNF) and Nerve Growth Factor (NGF) Blood Levels in Patients with Acute Carbon Monoxide Poisoning—A Preliminary Observations. Przegl. Lek..

[107] Girard M., Carrier P., Loustaud-Ratti V., Nubukpo P. (2021). BDNF Levels and Liver Stiffness in Subjects with Alcohol Use Disorder: Evaluation after Alcohol Withdrawal. Am. J. Drug Alcohol Abuse.

[108] Heberlein A., Büscher P., Schuster R., Kleimann A., Lichtinghagen R., Rhein M., Kornhuber J., Bleich S., Frieling H., Hillemacher T. (2015). Do Changes in the BDNF Promoter Methylation Indicate the Risk of Alcohol Relapse?. Eur. Neuropsychopharmacol. J. Eur. Coll. Neuropsychopharmacol..

[109] Anders Q.S., Ferreira L.V.B., Rodrigues L.C.D.M., Nakamura-Palacios E.M. (2020). BDNF mRNA Expression in Leukocytes and Frontal Cortex Function in Drug Use Disorder. Front. Psychiatry.

[110] Ahmadiantehrani S., Barak S., Ron D. (2014). GDNF Is a Novel Ethanol-Responsive Gene in the VTA: Implications for the Development and Persistence of Excessive Drinking. Addict. Biol..

[111] Maier H.B., Neyazi M., Neyazi A., Hillemacher T., Pathak H., Rhein M., Bleich S., Goltseker K., Sadot-Sogrin Y., Even-Chen O. (2020). Alcohol Consumption Alters Gdnf Promoter Methylation and Expression in Rats. J. Psychiatr. Res..

[112] Koskela M., Piepponen T.P., Lindahl M., Harvey B.K., Andressoo J.-O., Võikar V., Airavaara M. (2021). The Overexpression of GDNF in Nucleus Accumbens Suppresses Alcohol-Seeking Behavior in Group-Housed C57Bl/6J Female Mice. J. Biomed. Sci..

[113] Heberlein A., Bleich S., Bayerlein K., Frieling H., Gröschl M., Kornhuber J., Hillemacher T. (2008). NGF Plasma Levels Increase Due to Alcohol Intoxication and Decrease during Withdrawal. Psychoneuroendocrinology.

[114] Heberlein A., Muschler M., Frieling H., Behr M., Eberlein C., Wilhelm J., Gröschl M., Kornhuber J., Bleich S., Hillemacher T. (2013). Epigenetic down Regulation of Nerve Growth Factor during Alcohol Withdrawal. Addict. Biol..

[115] Neupane S.P., Lien L., Ueland T., Mollnes T.E., Aukrust P., Bramness J.G. (2015). Serum Brain-Derived Neurotrophic Factor Levels in Relation to Comorbid Depression and Cytokine Levels in Nepalese Men with Alcohol-Use Disorders. Alcohol.

[116] Girard M., Malauzat D., Nubukpo P. (2019). Serum Inflammatory Molecules and Markers of Neuronal Damage in Alcohol-Dependent Subjects after Withdrawal. World J. Biol. Psychiatry.

[117] Wedekind D., Neumann K., Falkai P., Malchow B., Engel K., Jamrozinski K., Havemann-Reinecke U. (2010). S100B and Homocysteine in the Acute Alcohol Withdrawal Syndrome. Eur. Arch. Psychiatry Clin. Neurosci..

[118] Jahangir S., Allala M., Khan A.S., Muyolema Arce V.E., Patel A., Soni K., Sharafshah A. (2023). A Review of Biomarkers in Delirium Superimposed on Dementia (DSD) and Their Clinical Application to Personalized Treatment and Management. Cureus.

[119] Huang M.-C., Chen C.-H., Liu H.-C., Chen C.-C., Ho C.-C., Leu S.-J. (2011). Differential Patterns of Serum Brain-Derived Neurotrophic Factor Levels in Alcoholic Patients with and without Delirium Tremens during Acute Withdrawal. Alcohol. Clin. Exp. Res..

[120] Wyrobek J., La Flam A., Max L., Tian J., Neufeld K.J., Kebaish K.M., Walston J.D., Hogue C.W., Riley L.H., Everett A.D. (2017). Association of Intraoperative Changes in Brain-Derived Neurotrophic Factor and Postoperative Delirium in Older Adults. BJA Br. J. Anaesth..

[121] Brum C., Stertz L., Borba E., Rumi D., Kapczinski F., Camozzato A. (2015). Association of Serum Brain-Derived Neurotrophic Factor (BDNF) and Tumor Necrosis Factor-Alpha (TNF-α) with Diagnosis of Delirium in Oncology Inpatients. Rev. Bras. Psiquiatr. Sao Paulo Braz..

[122] Wang S., Greene R., Song Y., Chan C., Lindroth H., Khan S., Rios G., Sanders R.D., Khan B. (2022). Postoperative Delirium and Its Relationship with Biomarkers for Dementia: A Meta-Analysis. Int. Psychogeriatr..

[123] Shyam R., Solanki M., Patel M.L., Sachan R., Ali W. (2023). S100B as a Predictor of Delirium in Critically Ill Obstetric Patients: A Nested Case-Control Study. Int. J. Crit. Illn. Inj. Sci..

[124] de Alencar J.C.G., Garcez F.B., Pinto A.A.S., Silva L.O.J.E., Soler L.d.M., Fernandez S.S.M., Van Vaisberg V., Gomez Gomez L.M., Ribeiro S.M.L., Avelino-Silva T.J. (2023). Brain Injury Biomarkers Do Not Predict Delirium in Acutely Ill Older Patients: A Prospective Cohort Study. Sci. Rep..

[125] Saito T., Braun P.R., Daniel S., Jellison S.S., Hellman M., Shinozaki E., Lee S., Cho H.R., Yoshino A., Toda H. (2020). The Relationship between DNA Methylation in Neurotrophic Genes and Age as Evidenced from Three Independent Cohorts: Differences by Delirium Status. Neurobiol. Aging.

[126] Jockers-Scherübl M.C., Bauer A., Kuhn S., Reischies F., Danker-Hopfe H., Schmidt L.G., Rentzsch J., Hellweg R. (2007). Nerve Growth Factor in Serum Is a Marker of the Stage of Alcohol Disease. Neurosci. Lett..

[127] Dunne S.S., Coffey J.C., Konje S., Gasior S., Clancy C.C., Gulati G., Meagher D., Dunne C.P. (2021). Biomarkers in Delirium: A Systematic Review. J. Psychosom. Res..

[128] Niemelä O. (2023). Predictive Risk Markers in Alcoholism. Adv. Clin. Chem..

[129] Prokopieva V.D., Vetlugina T.P. (2023). Features of Oxidative Stress in Alcoholism. Biomeditsinskaia Khimiia.

[130] Stoicea N., McVicker S., Quinones A., Agbenyefia P., Bergese S.D. (2014). Delirium—Biomarkers and Genetic Variance. Front. Pharmacol..

[131] Shinozaki G., Braun P.R., Hing B.W.Q., Ratanatharathorn A., Klisares M.J., Duncan G.N., Jellison S.S., Heinzman J.T., Nagahama Y., Close L. (2018). Epigenetics of Delirium and Aging: Potential Role of DNA Methylation Change on Cytokine Genes in Glia and Blood Along With Aging. Front. Aging Neurosci..

[132] Ivanova D., Giannouli V. (2017). Lesch Type III Alcoholism in Bulgarian Women: Implications and Recommendations for Psychotherapy. Int. J. Caring Sci..

